# Observing the factors affecting fibrovascular regrowth after pterygium excision and comparing the efficacy and complications of conjunctival autograft with sutures versus fibrin glue

**DOI:** 10.22336/rjo.2023.47

**Published:** 2023

**Authors:** Arti Singh, Jagriti Rana, Anirrud Sharma, Srishti Nagarajan

**Affiliations:** * Department of Ophthalmology, Moti Lal Nehru Medical College, Prayagraj, India

**Keywords:** fibrovascular regrowth, conjunctival autograft, fibrin glue, pyogenic granuloma, corkscrew vessels, subconjunctival hemorrhage

## Abstract

**Aim:** To observe the factors affecting fibrovascular regrowth after pterygium excision and to compare the efficacy and complications of conjunctival autograft with sutures versus fibrin glue.

**Materials and methods:** 65 consenting patients with primary pterygium attending the outpatient department having appropriate indications for surgery were enrolled. Data was collected using personal interviews. Routine pre-operative ophthalmic examination was done, including visual acuity assessment, slit lamp examination, and fundus evaluation. Pterygium excision surgery was done on all patients using either Fibrin Glue or 10-0 nylon sutures. Patients were followed up at weeks 1, 4, 12, and 24 and any complications were duly noted.

**Results:** The fibrin glue group showed milder postoperative discomfort, symptoms, and signs compared to the suture group. Pyogenic granuloma (3.12%), corkscrew vessels (6.25%), and subconjunctival hemorrhage (24.99%) were more common in the fibrin glue group. FVG not crossing the limbus was observed in 6.25% of glue cases and 9.09% of suture cases, more in fleshy and large pterygia, while age and gender did not alter the incidence of FVG. No recurrences were observed in any group.

**Conclusion:** The incidence of fibrovascular regrowth (FVG) was not affected by age, gender, smoking, and surgical technique, but was positively correlated with length and grade of pterygium. The complication rate between the two groups was not found to be statistically significant. Despite causing severe postoperative discomfort and requiring prolonged surgical time, suture-assisted pterygium surgery is a cost-effective method still being used with long-term outcomes similar to fibrin glue.

## Introduction

The word pterygium is derived from the Greek word “pteryx”, which means wing. It is an ocular surface disease manifesting as a wing-shaped fibrovascular tissue that proliferates onto the cornea. It is characterized by cellular proliferation, inflammatory infiltrates with fibrosis, angiogenesis, and extracellular matrix breakdown.

Higher prevalence rates are observed in the tropics than in temperate latitudes, with the equatorial countries having a much higher prevalence rate (commonly referred to as the “pterygium” belt) [**1**]. Disease pathogenesis can be attributed to a genetic component, stem cell failure, extracellular matrix remodeling, cytokines, growth factors, and viral infections. There is a considerably higher risk in outdoor workers exposed to sunlight and hence to UV rays, especially in those working in the setting of highly reflective surfaces [**2**].

Despite the number of surgical and medical measures currently available, there is no consensus regarding the “ideal” treatment. A plethora of surgical techniques, like bare sclera excision, excision with conjunctival closure/transposition, and excision with antimitotic adjunctive therapies like laser therapy, beta radiation, thiotepa, and mitomycin-C have been described. High recurrence of up to 90% has been found with bare sclera technique and so, excision followed by ocular surface transplantation techniques like conjunctival autografting, amniotic membrane transplantation, etc., have gained widespread popularity recently [**3**]. Kenyon et al. introduced the technique of conjunctival autografting, which is now considered the best method since it has a very low recurrence rate (between 2% and 39%) and complications compared to other techniques [**4**]. It is therefore regarded as the procedure of choice for the treatment of both primary and recurrent pterygium [**3**]. Various methods are being used to secure the conjunctival graft in place, such as sutures, fibrin glue, and autologous blood. Many published studies are comparing the outcome of these techniques, in which few studies find them equivocal, while others found one technique better than the others [**5**-**8**].

This study was done to compare the results of conjunctival autograft with sutures versus fibrin glue and to observe the preoperative factors affecting the surgical outcome.

## Materials and methods

*Study population*

The study was conducted in the Department of Ophthalmology, M.D. Eye Hospital, M.L.N. Medical College, Prayagraj, India, over 1 year after obtaining local Ethical Committee clearance. 65 patients with primary pterygium who attended our outpatient department were enrolled in this study. We included patients with primary pterygium having any of these indications for surgery: encroachment upon the visual axis, foreign body sensation due to recurrent inflammation, or cosmetic concern.

Exclusion criteria were recurrent pterygium, double-headed pterygium, use of antiglaucoma medications, presence of immune system diseases, eyelid or ocular surface diseases (e.g. dry eye, blepharitis, Sjogren syndrome), and hypersensitivity to any component of fibrin glue.

After obtaining the informed consent, a personal interview was used to collect data from patients. Routine pre-operative ophthalmic examinations were done on all patients and included visual acuity assessment, slit lamp examination, and fundus evaluation.

The clinical grading of pterygium was done based on its length of encroachment over the cornea (measured from the limbus): Grade 1 -<2 mm, Grade 2 - 2-4 mm, Grade 3 ->4 mm. Pterygium was also classified based on Tan’s classification: Atrophic (T1) - visible episcleral vessels under the body of pterygium; Intermediate (T2) - partially visible vessels under the body of pterygium; Fleshy (T3) - obscured episcleral vessels under the body of pterygium [**9**].

*Fibrin glue preparation*

Tisseel fibrin sealant by Baxter, Vienna, was used in our study. Fibrinotherm was used for mixing and warming the components of the glue. A sealer protein concentrate (containing fibrinogen and clottable protein) was reconstituted in an aprotinin solution, while thrombin was reconstituted in a calcium chloride solution and these two were mixed thoroughly. A separate disposable syringe was used to withdraw each solution and instead of using a duploject injector, we first put one drop of fibrinogen over the raw area and then one drop of thrombin component over it, leading to the better mixing of components in appropriate proportions. A single kit could be used to operate on 5-6 patients and since the mixed glue could not be used beyond 4-6 hours, cases were pooled (3-4 cases were operated using a single kit). This provided a cost-effective procedure to the patients.

*Surgical technique*

Patients were randomized into two groups. Photographic documentation of every patient was done pre-operatively and post-operatively. In the first group, 32 patients underwent pterygium excision with conjunctival autografting using fibrin glue, while 10-0 nylon sutures were used for securing the graft in place in 33 patients. The same surgeon performed all the surgeries.

Peribulbar block using 2% lignocaine with 1:1 lakh epinephrine was given in all cases. Following all aseptic precautions, a wire lid speculum was applied. Pterygium excision was done by avulsion technique. The underlying tenon’s capsule was removed up to the bare sclera. Cautery was done minimally only when and where found necessary. A thin conjunctival graft was retrieved from the superotemporal bulbar conjunctiva, taking care to keep it tenon-free. The size of the graft harvested was slightly larger than the size of the bare sclera.1 drop of fibrinogen component (thicker component) was placed on the bare sclera followed by the thrombin component (thinner component), followed by flipping the graft onto the bare sclera with special attention to maintain the “limbus to limbus” and “stromal side down” orientation. Gentle pressure was then applied to milk out any excess glue and free conjunctival margins were pressed. Graft adhesion could be confirmed in 2 minutes.

In the second group of 33 patients, pterygium excision was done in the same way as the first group and 4-6 sutures (10-0 nylon) were used to secure the graft in place. The duration of surgery was calculated from the time of placing the lid speculum until its removal. Removal of sutures was done 1 week after surgery. 

*Follow-up*

The bandage was removed on 1st post-operative day and patients were given moxifloxacin-dexamethasone eye drops 4 times a day. Follow-up visits were scheduled in 1st week, 1st month, 3rd month, and 6th month postoperatively. Antibiotic-steroid drops were tapered depending on the resolution of inflammation, from 4 to 2 times per day in 2nd week, then to once per day in 1st month, and eventually stopped. Pterygium recurrence was defined as any fibrovascular regrowth that crossed the limbus.

Patients were evaluated for the following at every visit: Visual acuity (Snellen’s chart), Intra-ocular pressure (Goldmann applanation tonometer), patient’s discomfort level, slit lamp examination with photographs, inflammation, subconjunctival hemorrhage, corneal epithelial damage/scarring, graft edema/recession/loss and any other complications.

## Results

**Fig. 1:**
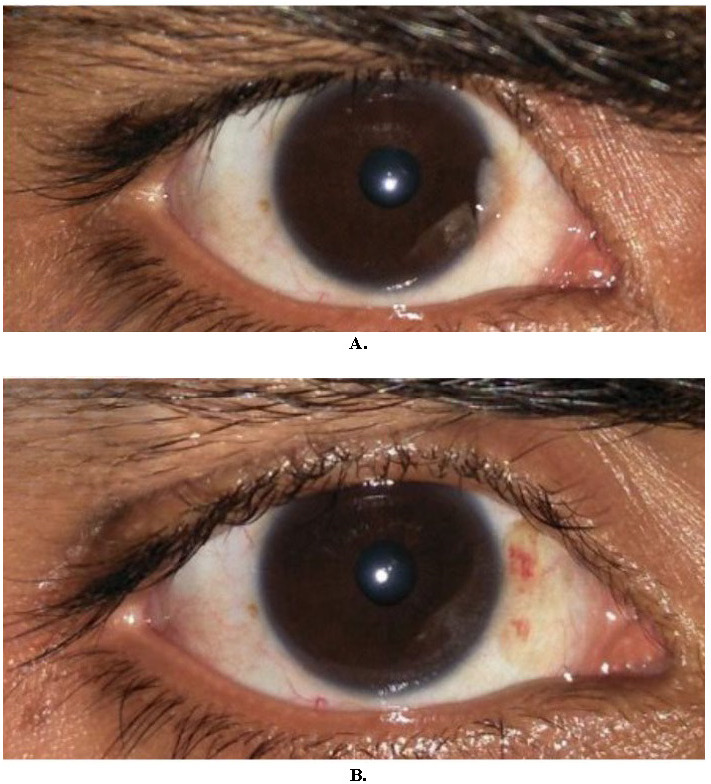
30 years/M with small atrophic pterygium, excised and grafted using fibrin glue (**A**). 1-month post-op (**B**)

**Fig. 2:**
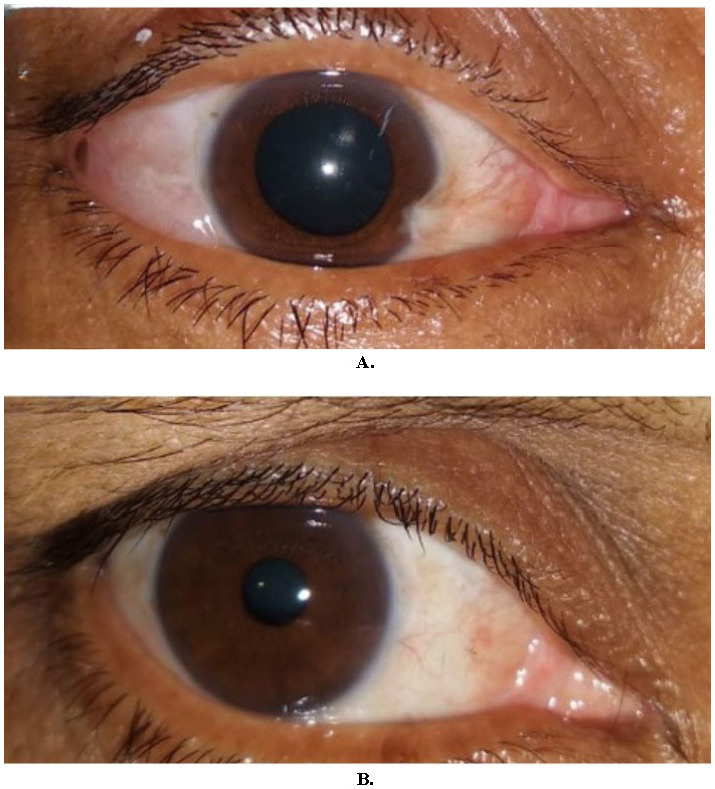
45 years/F with small atrophic pterygium, excised and grafted using 10-0 nylon sutures (**A**). 1-month post-op (**B**)

There were 16 males and 16 females in the group in which surgery was done with autologous fibrin glue. The mean age in this group was 40.95 years (± SD 13.59). The group that underwent surgery with 10-0 nylon sutures had 33 patients and consisted of 16 males and 17 females. The mean age in this group was 44.47 years (± SD 12.76) (**Fig. 1**,**2**).

In the fibrin glue group, the right eye was operated upon in 24 (75%) cases, whereas the left eye was operated upon in 8 (25%) cases. In the suture group, 20 (60.61%) cases were of the right eye, while 13 (39.39%) cases were of the left eye.

The clinical grading of pterygium was done based on the length of the pterygium tissue encroaching on the cornea, starting from the limbus. In the fibrin glue group, 14 (43.75%) cases were of grade1 (<2 mm),10 (31.25%) cases were of grade2 (2-4 mm), while 8 (25%) cases were of grade 3 (>4 mm). In the suture group, 8 (24.24%) cases were of grade 1, 19 (57.57%) cases were of grade 2, and 6 (18.18%) cases were of grade 3.

Morphological grading of pterygium was done based on Tan’s classification. In the fibrin glue group, 20 (62.5%) cases had atrophic (T1), 9 (28.12%) cases had intermediate (T2), and 3 (9.37%) cases had fleshy (T3) pterygium. In the suture group, 19 (57.57%) cases had atrophic, 11 (33.33%) cases had intermediate, and 3 (9.09%) cases had fleshy pterygium (**Table 1**).

**Table 1:** Demographic and clinical characteristics of patients in two groups

	Group A (fibrin glue)	Group B (suture)
No. of cases	32	33
Mean age (years)	40.95 ± 13.59	44.47 ± 12.76
Males	16	16
Females	16	17
		
RE	24 (75%)	20 (60.61%)
LE	8 (25%)	13 (39.39%)
		
Length of Pterygium (clinical grade)	Parameter	Parameter
<2 mm (grade 1)	14 (43.75%)	8 (24.24%)
2-4 mm (grade 2)	10 (31.25%)	19 (57.57%)
>4 mm (grade 3)	8 (25%)	6 (18.18%)
		
Grade of Pterygium		
Atrophic (T1)	20 (62.5%)	19 (57.57%)
Intermediate (T2)	9 (28.12%)	11 (33.33%)
Fleshy (T3)	3 (9.37%)	3 (9.09%)

The ratio of male to female was approximately 1:1 in both groups, suggesting no gender preponderance.

Diminution of vision was the main complaint of 14 patients (43.75%) in group A and 10 patients (30.30%) in group B. FB sensation was the complaint of 9 patients (28.12%) in group A and 12 patients (36.36%) in group B. 9 patients (28.12%) in group A had cosmetic concerns, while in group B there were 11 such patients (33.33%).

The operating time was significantly shorter in the fibrin group (19.28 ± 4.44 minutes), while in the suture group, it was 31.59 ± 2.33 minutes.

Generalized postoperative discomfort was observed in all cases. In the fibrin glue group, mild discomfort was observed in 26 cases (90.91%), while moderate discomfort was observed in 3 cases (9.09%). No patient had severe discomfort (0 cases). In the suture group, mild discomfort was observed in 3 cases (9.09%), moderate discomfort was observed in 28 cases (84.85%), and only 2 cases (6.06%) had severe discomfort. A statistically significant difference was found in postoperative discomfort between the two groups, which was greater in the suture group (P < 0.005).

Postoperatively, symptoms such as foreign body sensation, photophobia, and watering were assessed at every follow-up visit. In the fibrin glue group, 24 cases (75%) had mild symptoms, 8 cases (25%) had moderate symptoms, and none of the cases had severe symptoms. In the suture group, 2 cases (6.06%) had mild symptoms, 30 cases (90.91%) had moderate, whereas only 1 case (3.03%) had severe symptoms. Postoperative complaints were significantly higher in the suture group (P < 0.005).

Postoperative signs (Lid edema and Chemosis) were also assessed at each visit and were significantly higher in the suture group (P < 0.005). In the fibrin glue group, 26 cases (81.25%) had mild, 5 cases (15.62%) had moderate and no cases had severe postoperative signs. In the suture group, 2 cases (6.06%) had mild, 30 cases (90.91%) had moderate, while only 1 case (3.03%) had severe postoperative signs (**Table 2**).

**Table 2:** Postoperative symptoms and signs in two groups

	Group A (fibrin glue)	Group B (suture)		
POST-OP discomfort				
Mild	29 (90.91%)	3 (9.09%)		
Moderate	3 (9.09%)	28 (84.85%)		
Severe	0	2 (6.06%)		
				
Post-op symptoms (FB sensation/ photophobia/ watering)				
MILD	24 (75%)	2 (6.06%)		
MODERATE	8 (25%)	30 (90.91%)		
SEVERE	0	1 (3.03%)		
				
POST-OP signs (Lid edema/ Chemosis)				
Mild	26 (81.25%)	2 (6.06%)		
Moderate	5 (15.62%)	30 (90.91%)		
Severe	0	1 (3.03%)		
				
POST-OP Complications				
	N	%	N	%
Corkscrew vessels	2 (1 vessel observed)	6.25%	0	0%
Pyogenic granuloma	1	3.12%	0	0%
Corneal scarring	2 (grade 1)	6.25%	0	0%
Subconj. hemorrhage	Primary - 3, Secondary - 5 (after 1 week)	9.37%, 15.62%	Primary - 5, Secondary - 0	15.15%, 0%
Graft retraction	9	28.12%	1	3.03%
Recurrence	0	0%	0	0%
FVG not crossing limbus	2	6.25%	3	9.09%

**Fig. 3:**
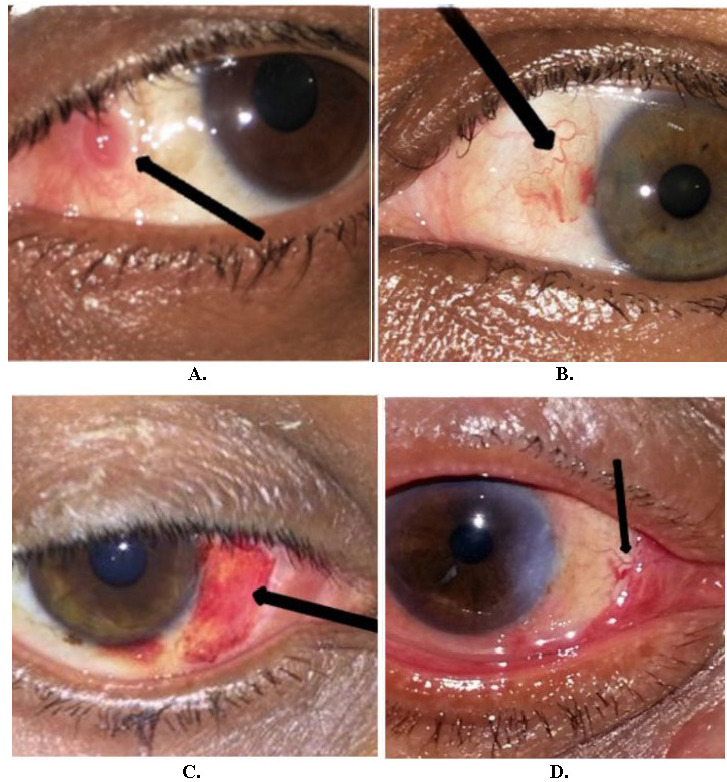
Postoperative complications. **A)** 35 years/M with pyogenic granuloma. **B)** 50 years/F with corkscrew vessel in graft. **C)** 28 years/M with grade 4 SCH at 1-week post-op. **D)** 55 years/M with grade 1 graft retraction on post-op day 1

Corkscrew vessels (**Fig. 3B**) were observed in 2 cases (6.25%) in the fibrin glue group, while none were observed in the suture group. Pyogenic granuloma (**Fig. 3A**) was only observed in 1 case (3.12%) in the fibrin glue group, and resolved after 2 months by giving cyclosporine eyedrops 0.05%. Grade 1 corneal scarring was observed in 2 cases (6.25%) in the fibrin glue group, while none of the cases in the suture group showed any corneal scarring.

Primary subconjunctival hemorrhage (SCH) was observed in 3 cases (9.37%), while secondary SCH (**Fig. 3C**) was observed in 5 cases (15.62%) in the fibrin glue group. In the suture group, primary SCH was observed in 5 cases (15.15%), while no cases of secondary SCH were present. Secondary hemorrhage was defined as subconjunctival hemorrhages, which were not there on the first postoperative day and were observed only after 1 week.

Among both groups, retrobulbar hemorrhage was observed in 1 case (3.03%) of the suture group, possibly as a procedural complication of peribulbar anesthesia.

Grade 1 graft retraction (**Fig. 3D**) was observed in 9 cases (28.57%) in group A and 1 (3.03%) case in group B. No graft loss was observed in either group. The most common site of graft retraction in our study was nasally (i.e. the graft retracted from its nasal edge towards the limbus, leaving the temporal part of the graft in place).

Fibrovascular growth was found in 5 patients, 2 in the glue group and 3 in the suture group. 1 FVG was observed in the intermediate type and 1 in the fleshy type in Group A. 1 FVG was observed in the intermediate type and 2 in the fleshy type in Group B.

Out of a total of 5 patients with FVG, 3 patients were of grade 3 pterygium, while 2 patients were of grade 2 pterygium. None of the patients with grade 1 pterygium showed any FVG.3 patients with FVG were < 50 years of age, while 2 patients were beyond 50 years of age. 

In our study, gender also seemed to play no role in determining the occurrence of FVG post-operatively, with 3 cases being males and 2 females.

Smoking was also analyzed as a separate factor affecting the occurrence of FVG. Only 1 smoker out of the 65 patients developed FVG, while 4 non-smokers also developed FVG (**Table 3**).

**Table 3:** Pre-operative factors affecting fibrovascular regrowth (FVG)

		Pre-operative factors affecting Fibrovascular regrowth		
Factor	Groups	Total no. of patients	No. of FVG	Percentage
AGE	< 50 years	47	3	6.38%
	≥ 50 years	18	2	11.11%
SEX	Male	33	3	9.09%
	Female	32	2	6.25%
				
LENGTH	<2mm	21	0	0%
	2-4mm	32	3	9.37%
	>4mm	12	2	16.67%
				
GRADE	Atrophic	39	0	0%
	Intermediate	20	3	15%
	Fleshy	6	2	33.33%
				
SMOKING	Smoker	11	1	9.09%
	Non-smoker	54	4	7.41%
				
SURGICAL TECHNIQUE	Fibrin Glue	32	2	6.25%
	Suture	33	3	9.09%

## Discussion

Several surgical techniques have been reported in the literature for pterygium. These include bare sclera excision, conjunctival and conjunctival-limbal autograft, and the use of an amniotic membrane. In addition, several adjunctive therapies, including the use of beta irradiation and mitomycin C (MMC), have been recommended due to their anti-fibrotic and anti-angiogenic properties [**10**]. Bare sclera closure was the most popular method for surgical removal of a primary pterygium from 1960 to the early 1980s. However, it has been shown that it is by far the least satisfactory method concerning recurrence rates (up to 80%) [**3**]. Conjunctival autografting gained popularity in the 1980s following the landmark article by Kenyon et al. in 1985, in which he reported a low recurrence rate of 5.3% using the conjunctival autograft technique [**4**]. 

In numerous studies, it has been observed that Limbal stem cell (LSC) plays a barrier role against conjunctival overgrowth on the cornea and its dysfunction may occur in pterygium. Conjunctivalization of corneal epithelium with overgrowth of fibrovascular tissue occurs due to deficiency of LSC at the limbus, and this holds for both primary and recurrent pterygium. The recurrence rate can be reduced by including these stem cells in conjunctival autografts [**11**]. While retrieving the graft, care should always be taken to include the limbal tissue and the LSCs.

Pterygium surgery with conjunctival autografting is now the procedure of choice in terms of efficacy and long-term stability. Even though conjunctival autografting is effective in preventing recurrence after pterygium surgery, suturing is a difficult task and requires surgical expertise and technical skill. Prolonged operation time during suture application is another problem for surgeons [**12**,**13**]. As a result, various other methods are being adopted to secure the graft, such as fibrin glue and autologous serum.

Fibrin glue is a biological and biodegradable material that induces minimal inflammation. In the early 1940s, fibrin glue was introduced in ophthalmology to fixate penetrating corneal grafts in rabbits. It is a biological tissue adhesive that imitates the final stages of the coagulation cascade. The first fibrin sealant approved by the FDA for use in the USA was Tisseel (Baxter Immuno, Vienna, Austria). In India it is available as Tisseel Fibrin Sealant (Baxter Vienna, Austria), and as Reliseal (from Reliance life-sciences).

Tisseel (Baxter, Vienna, Austria) consists of 2 component tissue adhesives mimicking natural fibrin formation. One of the components is fibrinogen mixed with factor XIII and aprotinin. The other component is the thrombin-CaCl2 solution. These components are mixed in equal amounts to prepare the glue. Thrombin acts on fibrinopeptides to break them into fibrin monomers, which then aggregate by cross-linking, leading to the formation of a fibrin clot.

Although in previous literature, in which male predominance was commonly observed, that could be attributed to greater outdoor activities of males, in our study, female patients were equal in number and it may be due to higher cosmetic concern observed in this gender.

In our study, more postoperative discomfort and inflammation in the form of congestion, chemosis, and lid swelling were found in the suture group as compared to the fibrin group. In the fibrin group, most patients reported mild inflammation, but it did not prove to be as bothersome to the patient as expected. All the complaints disappeared at the end of 6 months in either group. Similar to our study, Suzuki et al. [**14**] reported that sutures may sometimes cause inflammation of the conjunctiva and Langerhans cell migration into the cornea. Koranyi et al. [**12**] compared 7-0 vicryl suture to fibrin glue in their study and found that there was less patient discomfort and shorter operation time in the fibrin glue group. In their case series of 22 patients et al. [**13**] found that there were significantly fewer complaints in the fibrin group.

In our study, the mean operation time was found to be significantly shorter in the fibrin glue group. Farid et al. [**15**] compared fibrin glue with 8-0 vicryl, Srinivasan et al. [**5**], and Jiang et al. [**16**] with 10-0 nylon and they all observed similar results. 

Other advantages of fibrin glue use are the easy application of the graft, the possibility of using the graft with buttonholes, and the ease of surgery even in uncooperative patients. In our study, 1 fibrin glue was used for an average of 4 patients (1mL of fibrin glue is theoretically enough for 7-8 patients). Average operation cost also decreased in our study due to the pooling of cases scheduled for surgery.

In this study, subconjunctival hemorrhages were observed on the first postoperative day in 3 patients of the fibrin glue group (9.37%) and 5 patients of the suture group (15.15%). In the fibrin glue group, hemorrhage was found after 1 week in 5 patients (15.62%), which was not there on the first postoperative day. Secondary hemorrhages in these cases might be attributed to increased oozing of blood, while in the suture group, we made the graft bed more blood-free to facilitate suturing. All hemorrhages resolved within 3-4 weeks without any consequences.

Corkscrew vessels were found in 2 patients of the suture group. The presence of the corkscrew vessels indicated an increased rate of fibrosis and recurrences.

The recurrence rate of pterygium, which is done with conjunctival autografting technique with sutures, has been previously reported as 0-40%. In this study, we observed that no patient had a fibrovascular regrowth that crossed the limbus in the postoperative period. Koranyi et al. [**12**] found a recurrence of 8% in the fibrin group and 20% in the suture group following 6 months. In a study by Uy et al. [**13**], no recurrence was reported, but the limitation of the study was that their follow-up period was too short (2 months) to properly assess the recurrence rates. In a study by Dekaris et al. [**17**], in which they emphasized the limbal conjunctival autografting technique, no recurrence was recorded after a 6-month follow-up period. Contrary to these results, Baharet al. [**18**] found that the recurrence rate was higher in the fibrin group (11.9%) as compared to the suture group (7.7%). He hypothesized that it was possibly the fibrin coat that led to increased collagen accumulation and scar formation.

As far as pyogenic granulomas are concerned, it is postulated that abnormal vascular endothelial cell growth factors (VEGF), cytokine abnormalities, and fibroblast activation play a role in its pathogenesis although the exact mechanism remains unclear. Pyogenic granuloma found in our study at the retraction site can be attributed to increased inflammatory changes in the presence of a high amount of VEGF originating from the ischemic tissue damage site. Therefore, an operating surgeon should be wary of making maladapted conjunctival wound edges while using fibrin glue, as it can progress to fibrosis or recurrence.

Graft dislocation was not observed in either of the groups in our study. Previous literature also reports such graft dislocation (in which fibrin glue has been used) in very few cases. Missing grafts have been associated with a history of early removal of pressure patching (even though patients were instructed to leave it on overnight) and rubbing of eyes on the evening of the surgery. Mechanical forces can also alter the fibrin glue during the operation even if it sticks well. Thus, postoperative care in fibrin glue cases becomes even more important and must be well explained to the patients in detail.

Although rare, paravirus and prion infections with the fibrin group, remain an important issue of concern and so the patients should be well informed about the same before surgery. In our study, we found no anaphylaxis or occurrence of any infection in any patient during the follow-up period. Also, there is no report of any adverse reaction due to fibrin glue usage in previous literature, and the same was absent in our study.

We found that preoperative factors like age and gender had no significant effect on recurrences. Fibrovascular growth was more common in larger and fleshy pterygia and no fibrovascular growth was found in smaller and atrophic pterygia.

The major drawback of our study was the short duration of follow-up (6 months), which fell short of the time required (about 1 year) to demonstrate all complications and recurrences postoperatively. In most cases, recurrence was observed within 6 months, but it could also occur later. Another major drawback in our study was the absence of blinding. It was well known to the observer (who examined patients postoperatively through slit lamp biomicroscopy), which patients were glue cases and which were suture cases. This created a lacuna for the possibility of the observer being biased in favor of the fibrin glue group. Despite these shortcomings, the prospective nature of our study, surgeries performed by a single surgeon, and the inclusion of an adequate number of cases provided the necessary strength to our study.

## Conclusion

As compared to suture-assisted pterygium surgery, fibrin glue helped shorten the duration of the operation and reduce early postoperative inflammation. No statistically significant difference in complications was found between the two groups, although, graft retraction and pyogenic granuloma were observed more in the fibrin glue group. A longer follow-up is required to corroborate lower fibrovascular growth with fibrin glue use than suture use. Glue-assisted pterygium surgery causes less discomfort due to sutureless wounds as compared to suture-assisted pterygium surgery and so seems to be a promising alternative. Despite this advantage, cost is still a limiting factor in the use of fibrin glue in developing countries like ours. Despite causing severe postoperative discomfort and requiring prolonged surgical time, suture-assisted pterygium surgery is still being used, with long-term outcomes quite similar to fibrin. Age and gender have little impact on recurrences and large and fleshy pterygium are more prone to produce fibrovascular regrowth.

**Conflict of Interest statement**

The authors declare no conflict of interest.

**Informed Consent and Human and Animal Rights statement**

Informed consent has been obtained from all individuals included in this study.

**Authorization for the use of human subjects**

Ethical approval: The research related to human use complies with all the relevant national regulations, and institutional policies, is by the tenets of the Helsinki Declaration, and has been approved by the Ethical Committee of M.D. Eye Hospital, M.L.N. Medical College, Prayagraj, India.

**Acknowledgments**

None.

**Sources of Funding**

None.

**Disclosures**

None.

**Consent for publication**

According to ICMJE Recommendations for the protection of research participants.
